# Comparative Expression Analysis of Stress-Inducible Candidate Genes in Response to Cold and Drought in Tea Plant [*Camellia sinensis* (L.) Kuntze]

**DOI:** 10.3389/fgene.2020.611283

**Published:** 2020-12-23

**Authors:** Lidiia S. Samarina, Alexandr V. Bobrovskikh, Alexey V. Doroshkov, Lyudmila S. Malyukova, Alexandra O. Matskiv, Ruslan S. Rakhmangulov, Natalia G. Koninskaya, Valentina I. Malyarovskaya, Wei Tong, Enhua Xia, Karina A. Manakhova, Alexey V. Ryndin, Yuriy L. Orlov

**Affiliations:** ^1^Biotechnology Department, Federal Research Centre the Subtropical Scientific Centre of the Russian Academy of Sciences, Sochi, Russia; ^2^Institute Cytology and Genetics Siberian Branch of the Russian Academy of Sciences, Novosibirsk, Russia; ^3^State Key Laboratory of Tea Plant Biology and Utilization, Anhui Agricultural University, Hefei, China; ^4^Agrarian and Technological Institute, Peoples' Friendship University of Russia (RUDN University), Moscow, Russia

**Keywords:** gene expression regulation, homologs detection, genetic markers, principal component analysis, expression profile, candidate genes, abiotic stress, tea plant

## Abstract

Cold and drought are two of the most severe threats affecting the growth and productivity of the tea plant, limiting its global spread. Both stresses cause osmotic changes in the cells of the tea plant by decreasing their water potential. To develop cultivars that are tolerant to both stresses, it is essential to understand the genetic responses of tea plant to these two stresses, particularly in terms of the genes involved. In this study, we combined literature data with interspecific transcriptomic analyses (using *Arabidopsis thaliana* and *Solanum lycopersicum*) to choose genes related to cold tolerance. We identified 45 stress-inducible candidate genes associated with cold and drought responses in tea plants based on a comprehensive homologous detection method. Of these, nine were newly characterized by us, and 36 had previously been reported. The gene network analysis revealed upregulated expression in *ICE1*-related cluster of *bHLH* factors, *HSP70/BAM5* connected genes (hexokinases, galactinol synthases, *SnRK* complex, etc.) indicating their possible co-expression. Using qRT-PCR we revealed that 10 genes were significantly upregulated in response to both cold and drought in tea plant: *HSP70, GST, SUS1, DHN1, BMY5, bHLH102, GR-RBP3, ICE1, GOLS1*, and *GOLS3*. *SnRK1.2, HXK1/2, bHLH7/43/79/93* were specifically upregulated in cold, while *RHL41, CAU1, Hydrolase22* were specifically upregulated in drought. Interestingly, the expression of *CIP* was higher in the recovery stage of both stresses, indicating its potentially important role in plant recovery after stress. In addition, some genes, such as *DHN3, bHLH79, PEI54, SnRK1.2, SnRK1.3*, and *Hydrolase22*, were significantly positively correlated between the cold and drought responses. *CBF1, GOLS1, HXK2*, and *HXK3*, by contrast, showed significantly negative correlations between the cold and drought responses. Our results provide valuable information and robust candidate genes for future functional analyses intended to improve the stress tolerance of the tea plant and other species.

## Introduction

Cold and drought reduce the yield and geographical distribution of most horticultural crops worldwide. Both can lead to decreased water potential of tissues and induce reactive oxygen species accumulation, which causes severe damage to various cellular components (Minhas et al., 2017). Plant responses are complex, particularly in perennial woody crops, and hundreds of genes are involved in them (Chaves et al., 2003; Hao et al., 2018; Xia et al., 2019a). Earlier studies showed that plants have specific and non-specific responses to both stresses (Beck et al., 2007). Cold and drought induce common stress-inducible genes, while one of the stresses specifically induce some genes (Zhou et al., 2019). It is important to identify these common and unique responses under cold and drought stress for understanding the cross-talk mechanisms. To develop cultivars that are tolerant to both cold and drought, it is necessary to reveal the genes that are involved in both stresses and elucidate their response mechanisms to develop genetic markers that can help facilitate breeding programs (Minhas et al., 2017).

The tea plant (*Camellia sinensis* L.) is one of the most important economic crops in China, India, Sri Lanka, Kenya, and certain Caucasian countries (Turkey, Georgia, Russia, and Azerbaijan). This perennial woody evergreen crop is grown in more than 60 countries on five continents, from 49°N in Ukraine to 33°S in South Africa (Turkozu and Sanlier, 2017). Caucasus tea germplasm collection (44°36′40″ N, 40°06′40″ E) is located in the border region of the possible tea production and can be the source of the most tolerant cultivars; some genotypes here survive −15–17°C (Tuov and Ryndin, 2011). In most countries, tea plantations are affected by drought and cold stress that significantly reduces the yield and decreases the distribution of the crop in colder areas. Due to out-breeding and its long gestation period, the tea plant requires next-generation breeding strategies to improve its drought and cold tolerance through a deeper understanding of key regulators and their variants for precision introgressions to have better yield and quality under stress conditions. Therefore, efforts are needed to elucidate the global transcriptomic dynamics of multiple tea genotypes in drought and cold stress to critically discern key molecular players (Parmar et al., 2019).

Many transcription factors and metabolite-related genes have been shown to be involved in both the cold and drought responses of plants. For example, the key cold regulators *ICE, CBF*, and *DHN* transcription factors participate in both cold and drought and in other abiotic stresses (Liu et al., 2015; Liu S.-C. et al., 2016; Yin et al., 2016; Ban et al., 2017; Hu et al., 2020). The genes involved in the ABA-independent responsive pathway and the *bZIP*-mediated ABA-dependent pathway (Wang et al., 2012; Ban et al., 2017) also participate in tolerance to cold and drought. The overexpression of *CsbZIP6* in *Arabidopsis* resulted in hypersensitivity to several abiotic stresses (Cao et al., 2015). In addition, many other transcription factors (*WRKY, bHLH, NAC, HSP, LEA, CML*, and others) have been shown to be activated in tea plants in response to cold and drought (Yue et al., 2015; Wang Y.-X. et al., 2016; Chen et al., 2018; Cui et al., 2018; Ma et al., 2019). Recently, Li Y. et al. (2019) revealed that the genes *LEA2, HSP70, PRP, CIPs, PEIs, TLPs*, and *ChiA* were more strongly expressed under cold stress in tolerant cultivars than in susceptible cultivars. Recent transcriptomic data on tea plant showed that 12 TF families (*AP2/EREBP, bHLH, bZIP, HD-ZIP, HSF, MYB, NAC, WRKY*, zinc-finger protein TFs, *SCL, ARR*, and *SPL*) might play crucial roles in tea plant responding to drought (Liu S.-C. et al., 2016). In *Arabidopsis thaliana*, forty three transcription factor families (primarily, *WRKY, NAC, MYB, AP2/ERF*, and *bZIP*) were found to regulate 56% of common genes expressed in drought and cold stress (Sharma et al., 2018).

However, we continue to lack a complex picture of the interactions between the core network and their downstream-regulated target proteins. Additionally, comparison of molecular profiles of an organism under different stresses would make it possible to identify the conserved stress mechanisms (Amrine et al., 2015; Muthuramalingam et al., 2017; Chamani Mohasses et al., 2020). Thus, we have to continue searching for new evolutionarily conserved and species-specific genes related to the stress response. In this study, we combined literature data with interspecific transcriptomic analyses (*A. thaliana* and *Solanum lycopersicum*) to select genes that are related to cold tolerance. We built a network of candidate genes to reveal their interactions with the corresponding homologs for *A. thaliana*. We phenotypically screened a panel of Caucasian tea genotypes for cold and drought tolerance. Further expression analyses of 45 genes were performed in the most tolerant genotype under long-term stress induction and during the following recovery. The cold and drought expression profiles for each gene were compared to analyze overlapping responses in tea plant to both stresses, and correlations between cold and drought were revealed. Our results provide valuable information and robust candidate genes for future functional analyses intended to improve the stress tolerance of the tea plant and other species.

## Materials and Methods

### Candidate Genes Selection

To evaluate the cross-talk of the genetic response between cold and drought, cold responsive genes were selected as described below. The same genes have been tested in response to drought conditions.

We performed the interspecific analysis of transcriptomic data from the NCBI GEO database (ncbi.nlm.nih.gov/geo/, Barrett et al., 2012) for revealing candidate genes with increasing expression during cold. Using the datasets GSE103964, GSE112225, GSE116964 for *A. thaliana* and GSE78154 for *S. lycopersicum* the fold changes of gene expression under cold were calculated and ranks of genes were assigned according to their upregulation quartile (from 1 to 4) (Supplementary Table 1). Next, we compared top quartile genes between *A. thaliana* and *S. lycopersicum* using standalone BLAST (Camacho et al., 2009). As a result, nine orthologs were detected as genes with the highest rank in both species, and their nine corresponding orthologs of *Camellia sinensis* were added in experiment.

Further corresponding homologs in tea plant were characterized using BLAST against the Tea Plant Information Archive database (Xia et al., 2019b, Supplementary Table 2). The corresponding *A. thaliana* orthologs of *C. sinensis* were also identified from Li Y. et al. (2019) using the best-scored BLAST result. The selected genes (Table 1, Supplementary Table 2) were further annotated by the blast to the *A. thaliana* TAIR database (Lamesch et al., 2012). Primers were designed using PrimerQuest (eu.idtdna.com/Primerquest) with default parameters and amplicon size between 100 and 250 bp. The quality of the primers was revised using service Multiple Primer Analyzer by Thermofisher Scientific and PCR electrophoresis.

**Table 1:** Candidate cold responsive genes in tea plant.

**Source**	**Gene ID**	**Description**	**Trivial name**
Best hit of interspecies top ranked genes	TEA003328	Galactinol synthase 1	*GOLS1*
	TEA006793	Galactinol synthase 3	*GOLS3*
	TEA030611	Glycine-rich RNA-binding protein 3	*GR-RBP3*
	TEA021045	Endotransglucosylase	*HYDROLASE 22*
	TEA020473	Responsive to high light 41	*RHL41*
	TEA010353	Calcium underaccumulation 1	*CAU1*
	TEA003997	Pectin methylesterase 41	*PME41*
	TEA004079	Dehydration response element-binding protein 26	*DREB26*
	TEA024722	Aba- and osmotic-stress-inducible	*ARCK1, CRK45*
Upregulated cold responsive gene (Li Y. et al., 2019)	CSA032195	G-type lectin S-receptor-like serine/threonine-protein kinase At4g27290 [*Vitis vinifera*]	*GsSRK*
	CSA031147	G-type lectin S-receptor-like serine/threonine-protein kinase RKS1[*Theobroma cacao*]	*GsSRK1*
	CSA001565	LRR receptor-like serine/threonine-protein kinase FLS2-like	*FLS2*
	CSA020614	Receptor-like serine/threonine-protein kinase ALE2 [*Nicotiana sylvestris*]	*RPK2*
	CSA000608	Ethylene-responsive transcription factor ERF021[*Arabidopsis lyrata* subsp. Lyrata]	*AP2/ERF-AP21*
	CSA000348	Ethylene-responsive transcription factor SHINE 2-like [*Cucumis melo*]	*AP2/ERF-ERF2*
	CSA034862	Ethylene response factor 6	*AP2/ERF-ERF6*
	CSA023474	Bhlh transcription factor bhlh102	*bHLH102*
	CSA033910	Probable WRKY transcription factor 42	*WRKY42*
	CSA002423	PREDICTED: zinc finger CCCH domain-containing protein 30 [*Ricinus communis*]	*ZAT30*
	CSA003726	Late embryogenesis abundant protein 3L-1 [*C. sinensis*]	*LEA3*
	CSA031822	Late embryogenesis abundant protein [*C. sinensis*]	*LEA2*
	CSA012537	Heat shock 70 kda protein, mitochondrial-like	*HSP70*
	CSA014200	36.4 kda proline-rich protein-like [*Malus domestica*]	*PRP*
	CSA016010	Putative cold-inducible protein [*C. sinensis*]	*CIP*
	CSA001876	Probable pectinesterase/pectinesterase inhibitor 54	*PEI54*
	CSA035791	Endoglucanase 11-like [*Jatropha curcas*]	*EGase11*
	CSA000129	thaumatin-like protein 1b	*TLP1*
	CSA028426	Peroxidase 73 [*Vitis vinifera*]	*POD73*
	CSA006422	Glutathione S-transferase [*Camellia japonica*]	*GST*
	CSA010521	Beta-amylase 5 [*C. sinensis*]	*BMY5*
	CSA000011	Sucrose synthase 1 [*C. sinensis*]	*SUS1*

*The genes are retrieved from the TPIA database and the study from Li Y. et al. (2019). For detailed information please see Supplementary Table 2*.

#### Analysis of Relevance of Selected Genes and Their Interactions

A combined scored method was used to rank the identified genes from 1 to 9 points. In particular, we valued from 2 to 4 if genes have GO terms related to cold response [GO:0009409 Response to cold (“4”), GO:0006979 response to oxidative stress (“3”), GO:0050896 response to a stimulus (“2”)]. Also, we added a score from 1 to 4 if corresponded ortholog was detected in an upregulated cluster according to *A. thaliana* and *S. lycopersicum* data. Finally, we added 1 point if the gene was presented in related articles. Therefore, genes were ranked (Supplementary Table 2) from 1 to 9 points using a combined criterion.

#### Gene Network Reconstruction and Layout

The data from the literature sources and transcriptome analysis (see Supplementary Table 2) were used for the gene network reconstruction. Since most of the data for plant protein-protein interactions were obtained for *A. thaliana*, we identified the best-hit orthologs for *Arabidopsis* (Supplementary Table 2, column “AT ID”) and used them as source for building the corresponding gene network.

The network was reconstructed using the String database (https://string-db.org; Szklarczyk et al., 2019) with the following attributes: Textmining/Experiments/Databases interactions and threshold of interaction score = 0.15. For further layout and visualization, we used the Cytoscape (cytoscape.org; Shannon et al., 2003) and algorithm Radial Layout by yFiles.

### Plant Material

**Figure 1:**
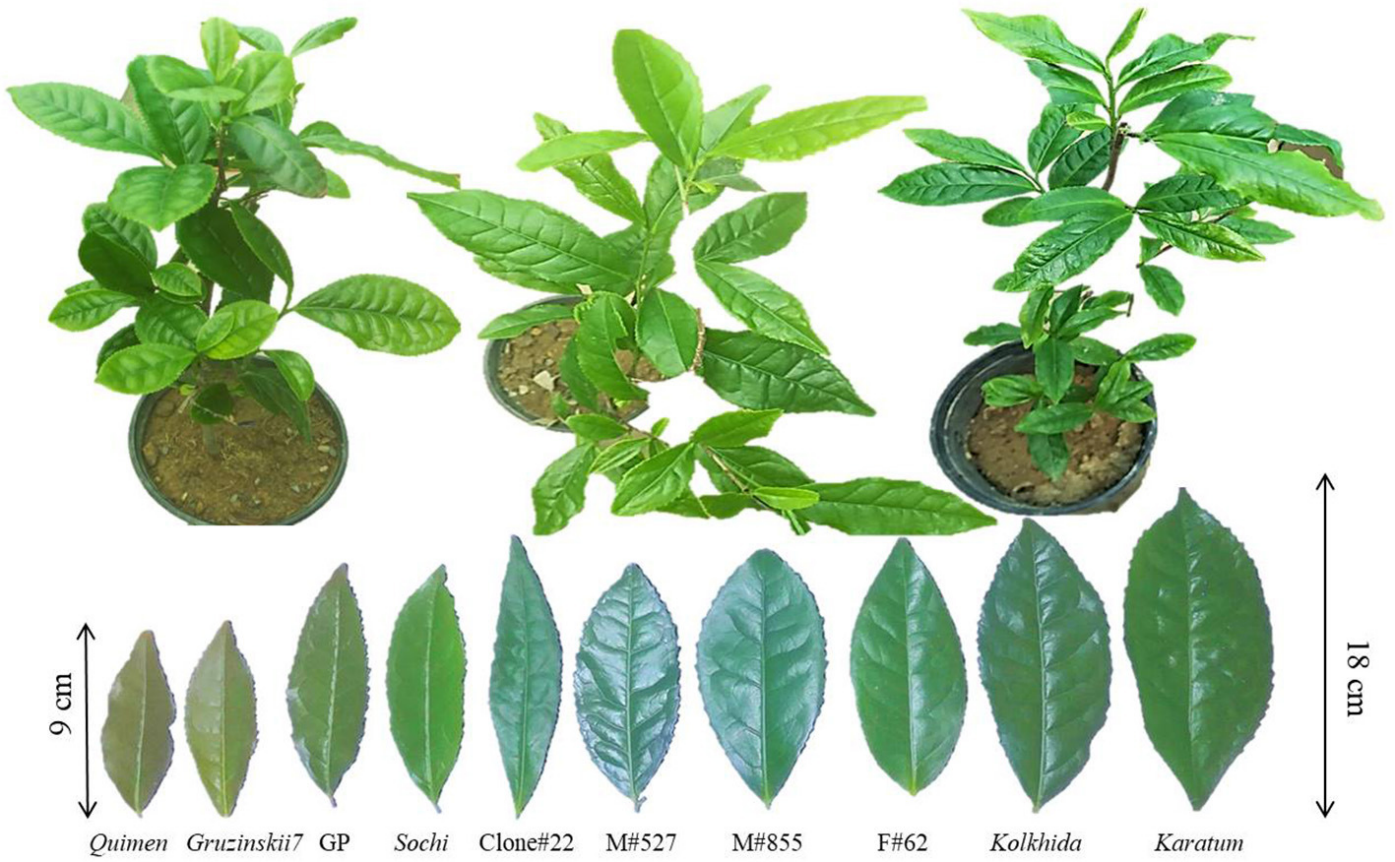
Three-year old tea plants used for the cold and drought treatments. Pot diameters−20 cm, plant heights 40–55 cm.

Three-year-old plants of ten elite tea genotypes obtained by vegetative propagation in FRC SSC RAS (Federal Research Center the Subtropical Scientific Center of the Russian Academy of Sciences) were used for leaf samplings. Ten genotypes of the local breeding were included in this study: *Quimen, Gruzinskii7*, GP, *Sochi*, Clone#22, M#527, M#855, Form#62, *Kolkhida, Karatum*. Among them, *Quimen, Gruzinskii7* were earlier showed to be the cold- and drought-tolerant genotypes; *Kolkhida* and *Karatum* were earlier showed to be cold-susceptible and drought-susceptible genotypes. Other clones and mutant forms showed medium cold-tolerance of drought-tolerance (Gvasaliya, 2015). Plants were grown in 2-liter pots filled with brown forest acidic soil (pH = 5.0) (Figure 1). Only healthy plants were selected for these experiments. Ten plants of each genotype were included in the study. For each assessed parameter, 2nd, 3rd, and 4th mature leaves were used for samplings. Experimental treatments with these plants were replicated twice in the period 2019 to 2020.

### Stress Induction and Phenotypical Screening for Tolerance

Control treatment: Before the stress treatments, plants were grown for 3 months in control conditions with the temperature of +22–25°C (with an illumination regime of 16 h of light and 8 h of dark, with the light intensity of 4000 lux with normal irrigation).

Cold treatment: Cold stress was induced in cold chambers HF-506 (Liebherr, Denmark) as follows: decreasing the temperature by 0- +2°C for 10 days to reveal the cold acclimation responses. After that, the temperature was gradually increased to +10°C during 10 days (Recovery-Cold treatment). Drought treatment: Drought stress was induced in a laboratory climatic chamber by gradually decreasing the watering till 15–17% of water content in soil (comparing with control 28–30%) during 10 days (drought treatment) to reveal the drought acclimation response. After that, watering was gradually increased until 28–30% for 10 days (Recovery-Drought treatment). During the treatments, the illumination regime was the same as in the control conditions.

For phenotypical evaluation of the tolerance to stress relative electrical conductivity was measured before the stress induction and after the stress inductions. Relative electrical conductivity was measured using a portable conductivity meter ST300C (Ohaus) to assess the electrolyte leakage indicating the damage of leaf tissues. The leaf sample was immersed in 150 ml of deionized water. The measurement of electrical conductivity was done immediately after immersion (L1) and 2 h later (L2). The relative electrical conductivity (REC, %) was calculated as: $REC=\frac{L1}{L2}*100$ (Bajji et al., 2001).

### Gene Expression Analysis

Total RNA was extracted from the third mature leaf in three biological replicates by the CTAB method (Doyle and Doyle, 1991) with minor modifications. The concentration and quality of RNA were determined using BioDrop μLite spectrophotometer and integrity was assessed by agarose gel electrophoresis. RNA samples were treated with DNase I and reverse transcription was performed using the MMLV-RT kit (Biolabmix, Russia). The efficiency of DNaseI treatment and reverse transcription were tested by agarose gel electrophoresis and by qRT-PCR. The results of this verification were evaluated by the presence/absence of a PCR product in RNA samples before and after DNaseI treatment, and by observing the size of PCR fragments in RNA samples before treatment and its cDNA synthesis. Only those samples that confirmed the absence of genomic DNA contamination were included in further analysis of gene expression. *Actin* (F: 5′-CCATCACCAGAATCCAAGAC-3′; R 5′-GAACCCGAAGGCGAATAGG-3′) (Hao et al., 2014) was taken as a reference gene and results were quantified using a Light Cycler 96 analyzer (Roche, Japan). The relative gene expression level was calculated by the Livak and Schmittgen (2001) using the following algorithm: 2^−ΔΔCq^, where:

$$\begin{array}{l} \Delta \Delta \text{Cq}={\left({\text{Cq}}_{gene\;of\;interest}-{\text{Cq}}_{internal\;control}\right)}_{treatment}\\ -{\left({\text{Cq}}_{gene\;of\;interest}-{\text{Cq}}_{internal\;control}\right)}_{control} \end{array}$$

### Statistical Analysis

All analyses were repeated twice with three biological replications in each. Statistical analyses were carried out using XLSTAT software. Student t-test, principal component analysis, and Pearson's correlation tests and Wards-clusterization were performed to evaluate data and confirm the significant differences (at the level *p* ≤ 0.05) between the genes expression profiles and respective treatments.

## Results

### Reconstruction of the Cold Stress Response Gene Regulation Network in Tea Plant for Selection of Priority Targets for Experimental Expression Profiling

**Figure 2:**
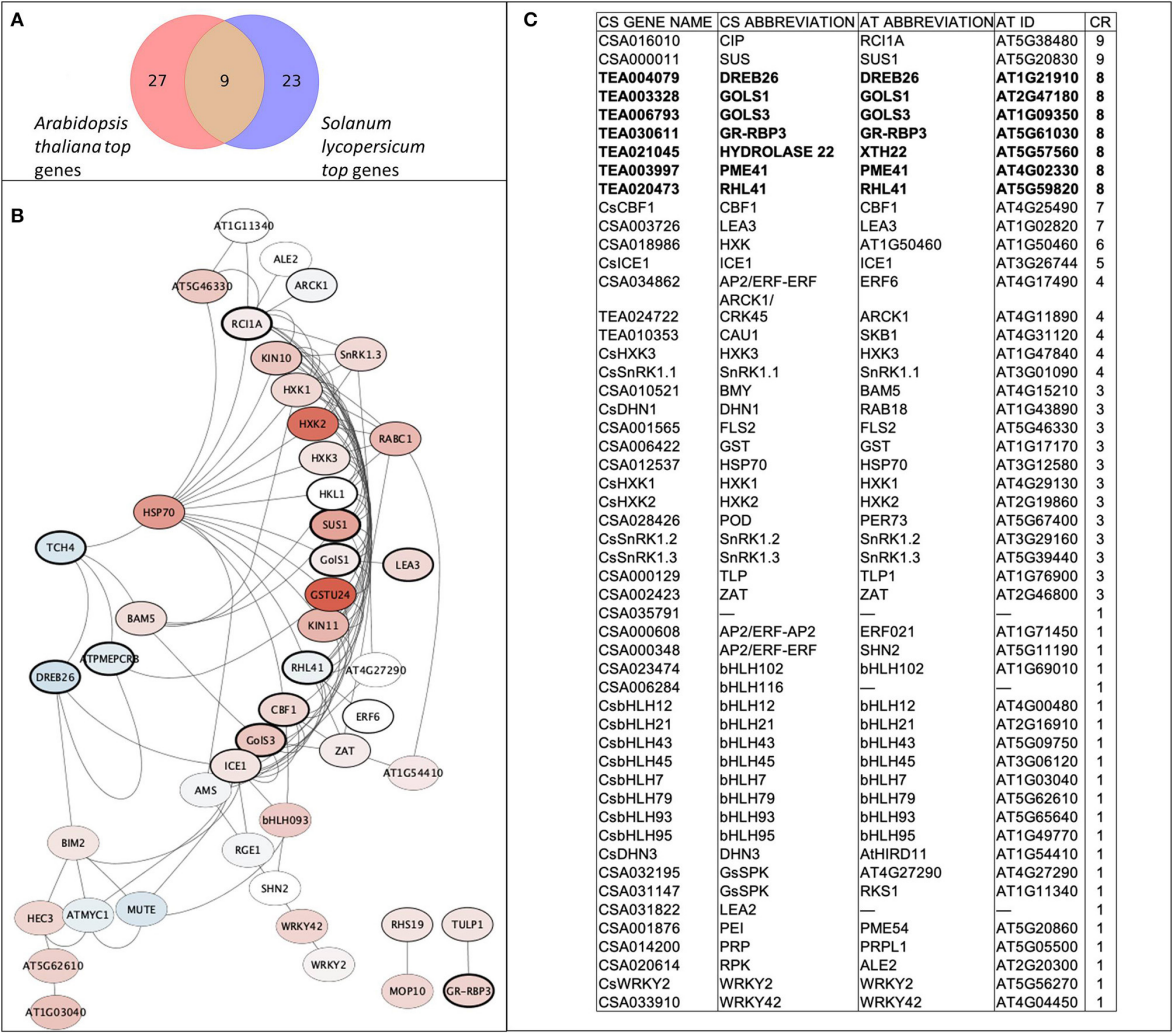
Core gene network of the stress-involved candidate genes. **(A)** Venn diagram of matched orthologs between top quartile of upregulated genes from transcriptomic analysis; **(B)** Reconstructed gene network using corresponding orthologs genes of *A. thaliana*. Red color gamut refers to upregulation by experimental data during cold treatment, blue color gamut refers to downregulation. Thickness of node border is proportional to combined score of gene. **(C)** Table of gene network legend and matches between CS (*C. sinensis*) and AT (*A. thaliana*) genes sorted by their combined rank (CR) score.

A set of 52 genes was involved in the analyses, including nine *de novo* predicted genes from transcriptomic data analyses and 43 from recent articles related to the cold tolerance of *C. sinensis*. The following genes were drawn from the literature: *bHLH* factors (9), *GsSRK* (2), *SnRK1* (3), *HXKs* (3), *ERF* (3), *WRKY* (2), dehydrins (2), late embryogenesis abundant proteins (2), and others (*CBF1, ICE1, ZAT, HSP70, PRP, CIP, PEI54, TLP, POD, GST, BMY, ALE2*, and *FLS2*). In addition, using interspecies transcriptome analyses we stressed nine orthologs that were highly upregulated in both species (*A. thaliana* and *S. lycopersicum*) using cold treatment: two galactinol synthases (*GOLS1* and *GOLS3*), glycine-rich RNA-binding protein 3 (*GR-RBP3*), xyloglucan endotransglucosylase/hydrolase protein 22 (*XTH22, Hydrolase22*), zinc finger protein *RHL41*, histone methylase *SKB1*, pectinesterase inhibitor *PME41*, dehydration response element-binding protein *DREB26*, and protein kinase superfamily protein *ARCK1*. For a better overall understanding of the interactions and to verify our chosen gene set, the gene network was reconstructed using *A. thaliana* data (Figure 2). The core gene network was classified using the three indicated clusters and had 42 genes with 111 edges between them, which indicate their tight interconnection. Interestingly, 30 of 46 genes were upregulated, and seven genes were downregulated.

### Phenotypical Selection of Tolerant Genotype Under Cold and Drought Treatments

**Figure 3:**
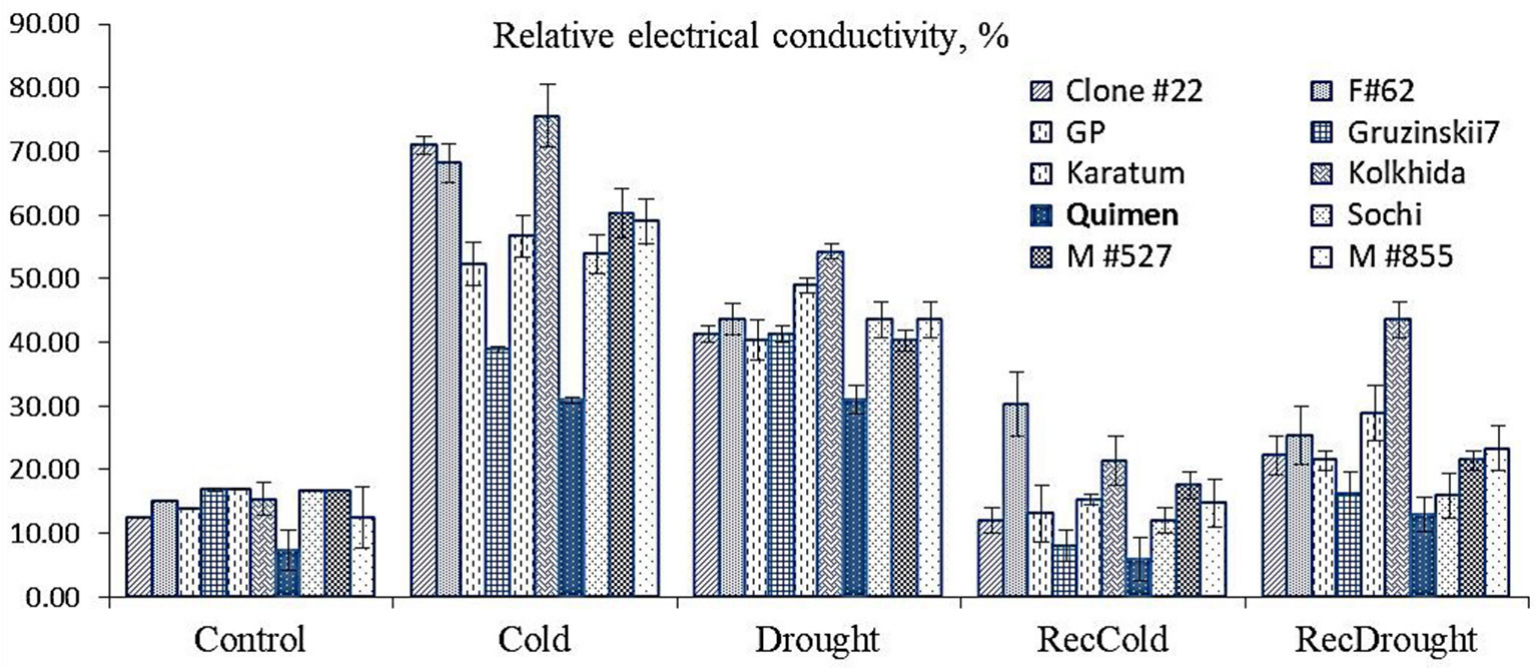
Relative electrical conductivity of leaf tissues during stress induction (dark blue color–tolerant cultivar selected for gene expression analysis) in ten tea germplasm accessions.

Cold resulted in increased relative electrical conductivity (REC) that reached 50–60% in most genotypes. Maximum REC was observed in three genotypes: Clone#22, Form#62, and cultivar Kolkhida. The lowest REC was observed in two cold-tolerant genotypes, Gruzinskii7 and Quimen, at 39 and 31%, respectively. Drought stress resulted in increased REC, which reached 40–49% in most genotypes. The highest REC, 54%, was observed in cv. Kolkhida. The lowest REC, 31%, was observed in cv. Quimen. The recovery stage showed no significant differences among the ten genotypes. Thus, the lowest REC under drought and cold induction was observed in Quimen, indicating the lowest damage of leaf tissues under cold and drought stress (Figure 3). This cultivar showed a similar REC for cold and drought treatment, which produced equal damage to tissues in both stresses, so this cultivar was used as the tolerant one in further gene expression analyses.

### Relative Expression Levels of the Studied Genes in Response to Cold and Drought

**Figure 4:**
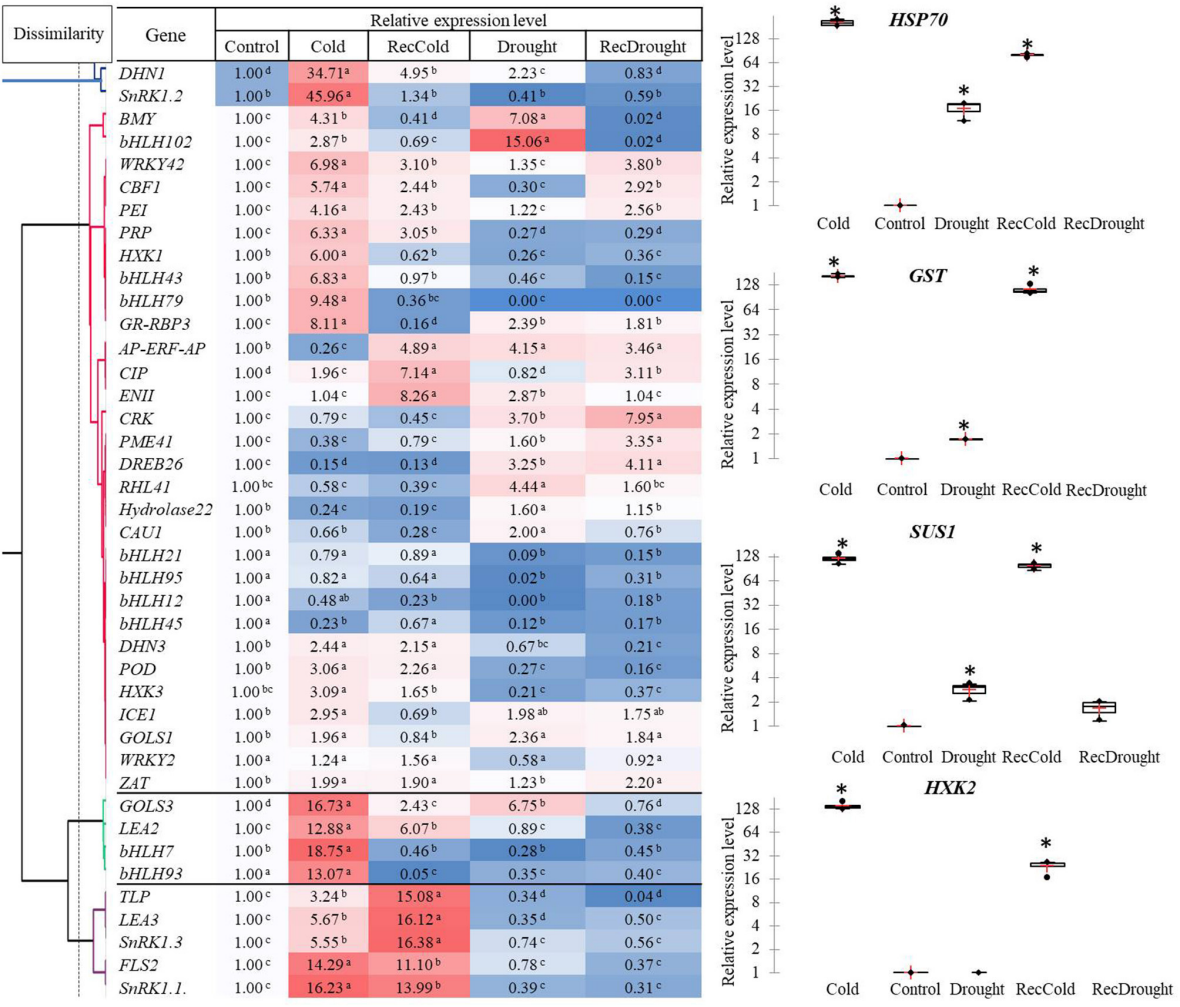
Heat map, hierarchical clustering and relative expression levels of studied genes in response to four treatments (Cold, Recovery-cold, Drought, Recovery-drought). The mean values of three replicates ± standard error (SE); asterisks and letters indicate significant differences at *P* < 0.05.

Of the 45 studied genes, the highest level of expression (hundred- fold) was observed in the four candidate genes in response to a given stress treatment: *HXK2* (Cold), *HSP70* (Cold, RecCold, Drought, and RecDrought), *SUS1*, and *GST* (Cold and RecCold) (Figure 4).

A heat map and hierarchical clustering revealed several clusters characterized by similar gene expression profiles (Figure 4). Cluster 1 combined the two genes *DHN1* and *SnRK1*. *2* with an over 30-fold induced expression in Cold. *DHN1* was also significantly upregulated in Drought and RecCold, indicating its importance in both stress responses.

The other two distant clusters with the most elevated expression were Cluster 3 and Cluster 4, including genes with a 10- to 19-fold upregulation in Cold and RecCold. Cluster 3 included the genes *GOLS3, LEA2, bHLH7*, and *bHLH93*, with a 13- to 19-fold upregulation under cold stress. Among these, *GOLS3* was also significantly upregulated in RecCold and Drought. Cluster 4 combined five genes (*SnRK1.1, SnRK1.3, LEA3, TLP*, and *FLS2*) that were significantly upregulated under Cold and RecCold, but no elevation in Drought or RecDrought was observed.

The most abundant cluster, Cluster 2, contained 30 genes separated into six sub-clusters. The first subcluster included two genes (*BMY5* and *bHLH102*) with the highest expression level in Drought (7- to 15-fold higher), and significantly induced expression in Cold (3- to 4-fold higher), and no elevated expression in recovery treatments. The second sub-cluster combined eight genes (*CBF1, PEI54, HXK1, bHLH43, bHLH79, WRKY42, PRP, GR-RBP*). These genes showed 3- to 9-fold upregulation in Cold. Of these, *WRKY42, CBF1*, and *PEI54* were significantly elevated in RecCold and RecDrought. In addition, four were downregulated in Drought and RecDrought: *PRP, HXK1, bHLH43*, and *bHLH79*. The third sub-cluster included eight genes (*AP-ERF-AP, EGASE11, CRK45, PME, DREB26, RHL, Hydrolase22*, and *CAU1*), which were significantly upregulated in Drought with 2- to 4-fold change, but most were not elevated in Cold. Four genes of the *bHLH* family composed the fourth sub-cluster and were characterized by decreased expression in most treatments: *bHLH12, bHLH21, bHLH45*, and *bHLH95*. *DHN3, POD73*, and *HXK3* combined in the fifth sub-cluster, with about a 2- to 3-fold greater expression under Cold and RecCold but very little expression in Drought and RecDrought. The last sub-cluster was formed by *ICE1, GOLS1, WRKY2*, and *ZAT* and showed 2-fold greater expression in Cold and Drought, as well as being slightly elevated in Recovery treatments.

In summary, the genes significantly upregulated in both Drought and Cold were *HSP70, SUS1, GST, DHN1, BMY5, bHLH102, GR-RBP3, ICE1, GOLS1*, and *GOLS3*, indicating that they may have important roles in both types of stress response. The genes that were specifically upregulated in Cold were *SnRK1.2, HXK1, HXL2, bHLH43, bHLH79, bHLH7*, and *bHLH93*. The genes that were specifically upregulated in Drought were *RHL41, CAU1*, and *Hydrolase22*. The transcripts of *CIP* were mostly accumulated in RecCold and RecDrought, and the transcripts of *PME41* were mostly accumulated in RecDrought indicating the possibly important role of these two candidate genes in plant recovery after stress. Generally, the cold response was more active in our study than the drought response. More genes with the highest expression levels were induced in response to cold than to drought.

### PCA Analyses and Correlations in Different Responses

**Figure 5:**
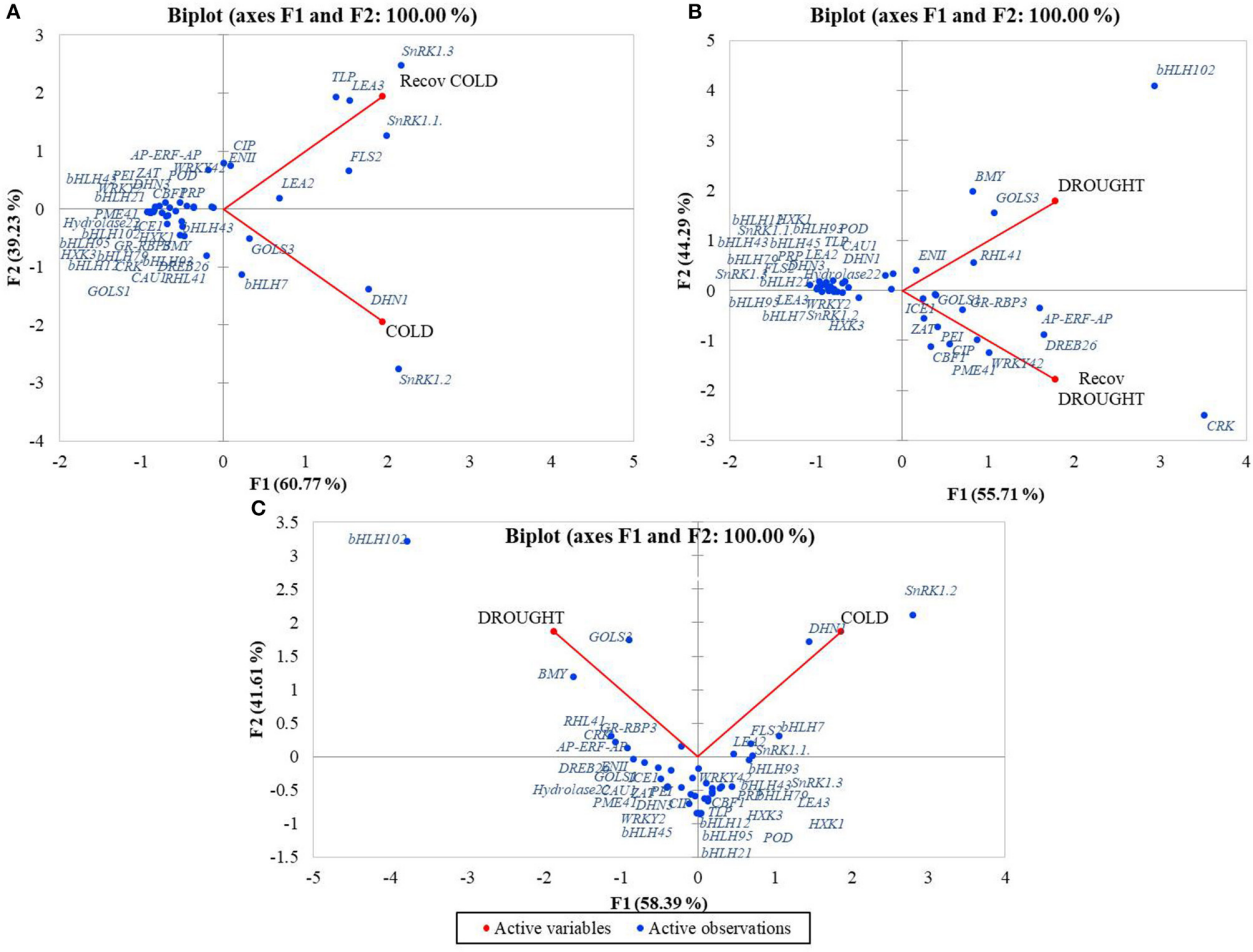
PCA analysis of expression profiles of candidate-genes distributed around treatments: **(A)** Cold/RecCold, **(B)** Drought/RecDrought, **(C)** Cold/Drought.

Pair comparison of treatments showed that the gene data points were clearly distributed between the two principal components Cold and RecCold. Most genes were densely grouped and showed similar expression profiles in Cold and RecCold, indicating a systemic response to cold stress. On the other hand, more genes were related to the principal component Cold. The RecCold cluster combined eight genes grouped distantly, which were strongly expressed in the recovery stage: *SnRK1*.*1, SnRK1*.*3, TLP, LEA2, LEA3, FLS2, EGase11*, and *CIP*. The genes *SnRK1*.*2, DHN1, GOLS3*, and *bHLH7* clustered distantly around the principal component Cold (Figure 5A).

The biplot Drought/RecDrought showed that most genes were densely grouped together with a similar expression pattern during both treatments. However, nine genes were distantly clustered around the RecDrought principal component: *CRK45, PME41, CBF1, CIP, PEI54, WRKY42, DREB26, ZAT*, and *ICE1*. Another eight genes were distantly clustered around the principal component Drought: *EGase11, RHL41, GOLS3, BMY5, bHLH102, DHN1, Hydrolase 22*, and *CAU1* (Figure 5B).

Finally, in the Cold/Drought biplot, most data points were clearly divided between the two principal components and showed the different characters of expression in the two stress responses. The two clusters with the greatest distances between Cold and Drought PCs were obtained. The first combined the six genes with the highest expression level in Drought: *GOLS3, BMY5, bHLH102, RHL41, CRK45*, and *GR-RBP3*. The second one combined the six genes with the highest expression level in Cold: *SnRK1*.*2, DHN1, FLS2, LEA2, SnRK1*.*1*, and *bHLH7*. Most of the other genes were also clearly divided between the principal components Cold and Drought (Figure 5C).

**Figure 6:**
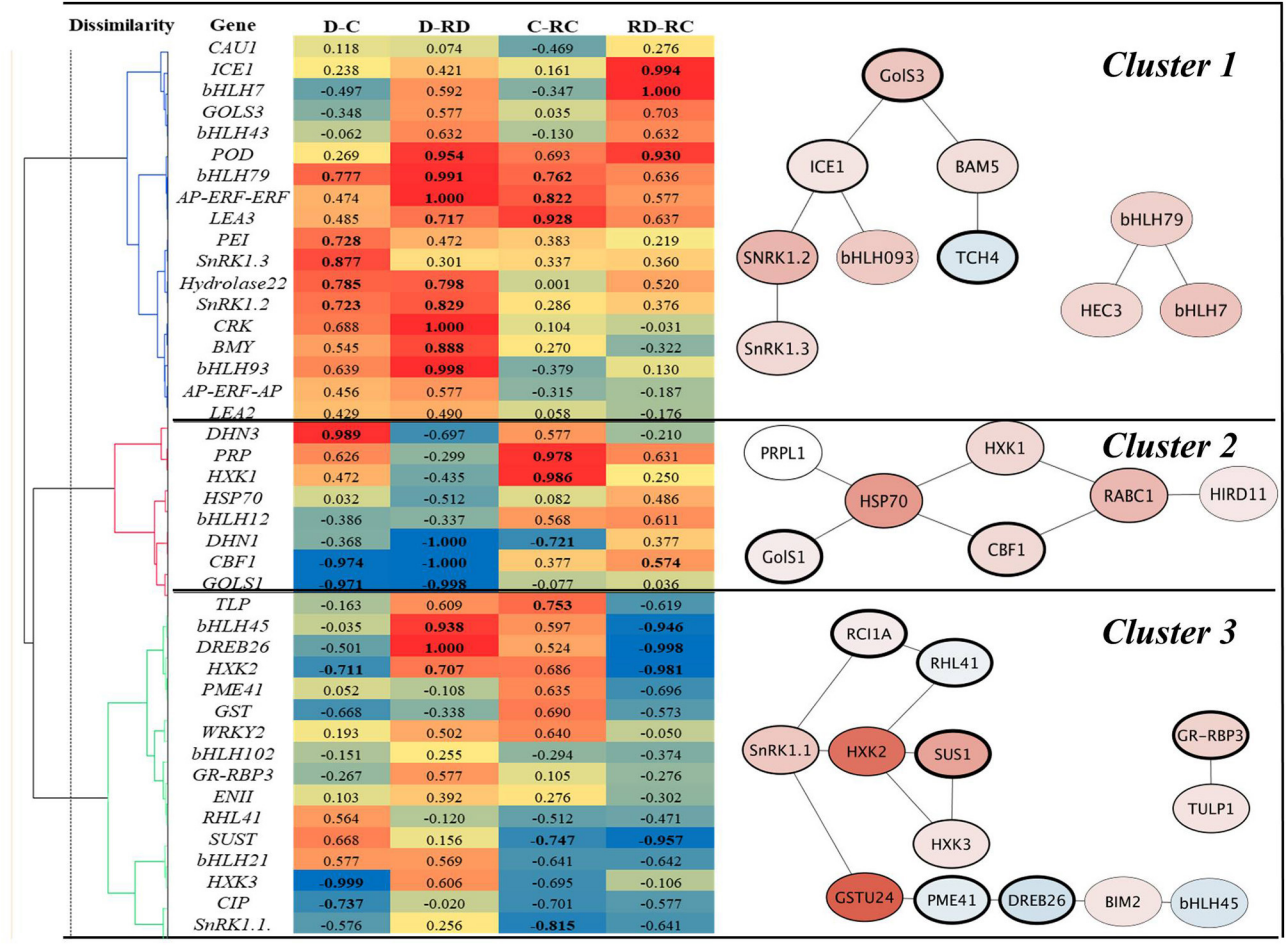
Correlation heat map and corresponding gene subnetworks (*A. thaliana*) of clusters marked by numbers 1–3. D-Drought, C–Cold, RD–Recovery drought, RC–recovery cold. Values in bold are different from 0 with a significance level alpha = 0.05.

The correlation analyses of responses to Drought, Cold, RecDrought, and RecCold resulted in three large clusters of candidate genes (Figure 6). The first, the largest cluster, included 18 genes with the highest positive and significant correlations between the treatments. This cluster combined three main subclusters. The first included the genes *ICE1* and *bHLH7*, which had a high positive correlation between RecCold and RecDrought. The second sub-cluster combined four genes that had a high positive correlation between Cold/RecCold and Drought/RecDrought: *POD73, bHLH79, AP-ERF-AP*, and *LEA3*. The third sub-cluster included genes with high positive correlations between Drought/Cold (*PEI54, SnRK1*.*2, SnRK1*.*3*, and *Hydrolase22*) and Drought/Recovery (*Hydrolase22, SnRK1*.*2, CRK45, BMY5*, and *bHLH93*).

The second large cluster combined nine genes. Of these, *DHN3* showed a positive correlation between Cold and Drought. *PRP* and *HXK1* showed a high positive correlation between Cold and RecCold. Three genes showed a strong negative correlation between Drought and RecDrought*: DHN1, CBF1*, and *GOLS1*. Additionally, *DHN1* was negatively correlated in Cold and RecCold; *CBF1* and *GOLS1* were negatively correlated in Drought and Cold.

The third big cluster combined 16 genes, divided into two big sub-clusters. One sub-cluster included eight genes, of which three showed a significant negative correlation between RecCold and RecDrought: *HXK2, DREB26*, and *bHLH45*. However, another three of these genes showed high positive correlations in Drought and RecDrought. Finally, the second small sub-cluster of Cluster 3 included six genes, of which four showed significant negative correlations between Cold and RecCold (*SnRK1*.*1* and *SUS1*) RecCold and RecDrought (*SUS1*), and Cold and Drought (*HXK3* and *CIP*).

In summary, the following genes were significantly positively correlated between Cold and Drought: *DHN3, bHLH79, PEI54, SnRK1*.*2, SnRK1*.*3*, and *Hydrolase22*. On the other hand, *CBF1, GOLS1, HXK2*, and *HXK3* showed significant negative correlations. Many genes were positively correlated between Drought and RecDrought, namely, *POD73, bHLH79, AP-ERF-ERF, LEA3, Hydrolase 22, SnRK1*.*2, CRK45, BMY5, bHLH93, bHLH95, DREB26*, and *HXK2*. Three genes showed negative correlations: *DHN1, CBF1*, and *GOLS1*. Six genes were positively correlated between Cold and RecCold: *bHLH79, AP-ERF-ERF, LEA3, PRP, HXK1*, and *TLP1*. Three genes were negatively correlated: *SnRK1*.*1, SUS1*, and *DHN1*. Finally, RecCold and RecDrought analyses resulted in four positively correlated genes (*ICE1, bHLH7, POD73*, and *CBF1*) and four negatively correlated genes (*bHLH45, DREB26, HXK2*, and *SUS1*).

## Discussion

### Reconstruction of the Cold Stress Response Gene Regulation Network in Tea Plant

To develop tolerant genotypes, breeders need reliable sets of informative genetic markers to select donors from germplasm collections. The homolog databases of candidate genes can be an efficient tool for finding these markers with *in silico* searches in model plant species. In our study, we used this approach to identify new possible candidate genes and their homologs in tea plants. We selected possible candidate genes and built a core network for 42 genes with 111 edges between them, which indicates their tight interconnection. *DREB26, GOLS1, GOLS3, GR-RBP3, Hydrolase22, PME41*, and *RHL41* are commonly found in *A. thaliana* and *S. licopersicum*. It is known that the evolutionary distance between *A. thaliana* and *S. lycopersicum* is very similar to distance between *A. thaliana* and *C. sinensis* (timetree.org). Thus, we proceeded from the assumption that nine identified genes may have a similar role for *C. sinensis*. Based on the constructed gene network we revealed that eight of the nine candidate genes are linked to the main network of the stress response. So it can be suggested that they belongs to the core part of the stress response, their functions are evolutionarily conservative and these genes can be predicted for the other plant species based on interspecific analysis. The hypothesis of the strong upregulation of galactinol syntases (*GOLS1* and *GOLS3*) and *GR-RBP3* and *DREB26* in tea stress responses was confirmed experimentally in our study (Figure 4).

We combined bioinformatics and experimental approaches to test nine new candidate genes that could be relevant for different plant species. However, among their orthologs in *C. sinensis*, only *GOLS3* and *GR-RBP3* were found to be upregulated during cold treatment. On the other hand, *CIP*, which has the highest score according to bioinformatics data (Figure 2), is highly upregulated during recovery. This can indicate a large difference between woody crops and grasses in responses to stress. In addition, well-known regulators such as *DHN1, HXK1, PEI*, and *CBF1* were confirmed to be highly upregulated during cold treatment. Therefore, experimental testing of well-known regulators with their new target genes for particular genotypes may be a useful and iterative approach for evaluating complex regulatory networks of stress adaptation in plants.

The gene-regulatory networks for cold and drought response remain an open topic for investigation due to the complex nature of genetic interactions and their genotype-specific character. For example, the divergence and specialization of gene networks involved in trichome development may be connected with the emergence of the plant taxa (Doroshkov et al., 2019). In our study, many regulators were connected to *HSP70* and tightly interconnected among each other. The *ICE1*-related *bHLH* cluster and *WRKY* factors were mostly upregulated, similarly to the *SnRK* complex, hexokinases, and galactinol synthases. However, *XTH22-PME41-DREB26*, and *bHLH12-bHLH45* were downregulated, which may indicate their coordinated repression.

### Phenotypical Selection of Tea Under Cold and Drought Treatments

North-Western Caucasus in Russia is the one of the northernmost regions of commercial tea growing in the World. Tea plantations in the region are not of a large scale, but the climate here is colder that is why tea growth without chemical plant protection because there is no pest and diseases. Seeds of tea plant were introduced to Caucasus in nineteenth century from China, Japan, India, Sri Lanka and Indonesia and represent a wide range of hybrid genetic diversity. Domestication of the tea plant in the Caucasus occurred within 150 years, during which the tea crop moved from the southern regions of Ozurgetti in Georgia (41°55′27″ N, 41°59′24″ E) to the Northern region in Maykop in Russia (44°36′40″ N, 40°06′40″ E) (Tuov and Ryndin, 2011). Tea breeding was conducted here from 1950th and as the result many local cultivars were developed, such as *Kolkhida, Qimen, Gruzinskii7, Karatum, Sochi*, and many others. Also the set of mutant forms such as M#527, M#855, F#62, Clone #22 and many others were developed by UV and chemical mutagenesis (Gvasaliya, 2015). The genotypes included in our study characterized by high yield and quality in the local conditions. Phenotyping of the tolerance was done using the common approach – the measurement of the relative electrical conductivity (see for example, Ban et al., 2017), that help to assess the electrolyte leakage caused by stress. The results confirmed that genotypes with large and thin leaf (for example, *Karatum, Kolkhida*) are less tolerant to cold and drought than the genotypes with small and thick leaf blades (such as *Quimen* and *Gruzinskii7*) (Figure 1). Our results on phenotypical evaluation correspond with the other studies on several plant species in which the drought-resistant genotypes showed tolerance to cold as well (Zheng et al., 2016; Lu et al., 2017; Li X. et al., 2019).

### Relative Expression Levels of the Studied Genes in Response to Cold, Drought and Recovery

#### Genes Upregulated in Response to Both Cold and Drought

In the tolerant genotype the expression levels of the genes *HSP70, DHN1, GST, SUS1, bHLH102, BMY5, GR-RBP3, ICE1, GOLS1*, and *GOLS3* were significantly higher in both Cold and Drought than in control, suggesting shared upstream pathways for signal transduction and regulation under these stimuli.

Among the nine *bHLH* genes included in this study, only *bHLH102* was increasingly expressed in both stress treatments, and we suppose that this new candidate gene can also be an important marker for abiotic stress tolerance in tea. In *A. thaliana*, this gene encodes positive brassinosteroid-signaling protein, and functional validation is necessary in tea plant.

The Hsp70s are highly conserved and widespread and important for protein folding, protein translocation, and the stress response in almost all subcellular compartments (Su and Li, 2008). The *HSP70* genes are upregulated in drought-tolerant Indian tea cultivars that are subjected to water stress (Maritim et al., 2016). In our study, the highest level of expression for *HSP70* (several hundred-fold) was observed in all experimental treatments (Cold, RecCold, Drought, and RecDrought) (Figure 3); however, it was more actively induced by Cold compared to Drought, indicating its possible importance in preventing the dehydration of cell compartments during low temperatures.

Another gene that was upregulated in cold and drought was *GST*. GSTs are a superfamily of enzymes that are notable for their role in phase-II detoxification reactions of quenching reactive molecules by adding glutathione (GSH) and protecting the cell from oxidative damage (Kumar and Trivedi, 2018). In previous work, *GST* and *POD* were upregulated in a tolerant tea cultivar under cold stress (Li Y. et al., 2019), which corresponds with our results. However, these genes were more strongly induced by cold, and we suppose that the cold response is characterized by stronger ROS-scavenging activity than the drought response.

The next gene with a multi-fold change in Cold and RecCold and significant upregulation in Drought was *SUS1*. It encodes sucrose synthase (Sus), a key enzyme of sucrose metabolism. Previous studies reported that the transcription levels of *Sus1* increased after exposure to cold and drought (Dejardin et al., 1999; Stein and Granot, 2019). However, based on the expression profile of *SUS1*, we speculate that sucrose–raffinose conversion is more strongly induced by cold than by drought in tea plant. Also, the bulk degradation of sucrose into glucose and fructose maybe a strategy employed by tea plants to double its osmotic contribution in response to severe drought and cold stress (Zheng et al., 2016).

Another new gene that was significantly overexpressed in response to both drought and cold was *GR-RBP3*, a class-IV GRP (RBP), which is involved in alternative splicing, transcriptional regulation, and developmental processes (Czolpinska and Rurek, 2018). Some GRPs have been described as proteins that mainly enhance plant tolerance to low temperatures. Here, we suppose that they may also be an important genetic marker of both cold and drought tolerance, with a functional role in the tea plant that it is necessary to clarify.

One more gene that was significantly upregulated during drought and cold stress was *BMY5*. BMYs degrade starch to soluble sugar, which leads to increased maltose, glucose, fructose, and sucrose levels after further conversion. We suggest that starch degradation is an important mechanism in tea, not only for cold tolerance but also for drought tolerance. This is consistent with the results recently published by Yue et al. (2019), who found that *BMY* genes contain many stress-related cis-acting elements, such as drought stress-related *ABRE, DRE1, MBS*, and *STRE*; cold stress-related *LTR*; and stress phytohormone-related *ERE* and *TCA*. Taken together, these results suggest that *BMY* genes are involved in the response of tea plants to multiple challenging environmental conditions and may be an important marker for the tea plant.

The last two genes that feature strong upregulation in response to drought and cold are *GolS1* and *GolS3*. GolS is a key enzyme in the synthesis of raffinose family oligosaccharides that function as osmoprotectants in plant cells. *GolS1*- or *GolS2*-overexpressing *Arabidopsis* has high intracellular levels of galactinol and raffinose in transgenic plants, which correlates with increased tolerance to drought and chilling stress (Panikulangara et al., 2004; Nishizawa et al., 2008; Li Y. et al., 2019). Our results support these findings and confirm that the mechanism of protecting salicylate from attack by hydroxyl radicals mediated by galactinol and raffinose is important for drought and cold defense.

#### Genes Specifically Upregulated in Drought

The genes specifically upregulated to a higher level in Drought were *RHL41, CAU1, Hydrolase22, CRK45, PME41* which suggests that these genes are conservative and may play vital specific roles in response to drought stress.

*RHL41*, which relates to the zinc-finger protein *Zat12*, is a representative of the small group of genes that respond similarly to many different environmental stresses (Iida et al., 2000; Davletova et al., 2005). A recent study of transgenic plants suggested that *Zat12* plays a role in different stress responses in *Arabidopsis* (Rizhsky and Liang, 2004; Vogel et al., 2005). Some authors have reported that *Zat12* acts as a suppressor of *CBF* transcription (Davletova et al., 2005; Vogel et al., 2005). We observed increased accumulation of *Zat12* (*RHL41)* transcripts during drought, indicating that this gene may have a specific function for drought stress responses in tea plant.

*CAU1* encodes an H4R3sme2-type histone methylase and acts as an immediate upstream suppressor of the *CAS* gene (encoding a putative Ca^2+^ binding protein that is proposed to be an external Ca^2+^ sensor). Elevated extracellular calcium decreases CAU1 protein levels and consequently the methylation level of H4R3sme2 in the CAS chromatin, thus derepressing *CAS* expression to close stomata (Fu et al., 2013). Our results indicate the specific activation of *CAU1* under drought. It may be that stomata closure mediated by *CAU1* is an important mechanism of defense against drought in tea plant. This corresponds with previous studies that have reported increased drought tolerance and stomatal closure in *cau1* mutants of *Arabidopsis* (Fu et al., 2013).

*Hydrolase22* was also specifically upregulated during drought stress. This gene encodes proteins that maintain the plasticity of the cell wall and increase its thickness by reinforcing the secondary wall with hemicellulose and lignin deposition (Le Gall et al., 2015). We thus consider that the adjustment to the cell wall mediated by this enzyme is an important mechanism in adaptation to drought in tea plant.

Different families of protein kinase had positive regulatory roles in responding to drought stress in tea plant, leading to maintain homeostasis of drought stress and water signal transduction (Liu S.-C. et al., 2016). Our result showed that *CRK45* was upregulated in Drought and RecDrought but not in Cold. It is a member of the membrane-anchored receptor-like protein kinases (RLKs), which recognize extracellular signals at the surface of the cell and activate a downstream signaling pathway by phosphorylating specific target proteins (Tanaka et al., 2012). CRKs make up a large subgroup of the RLKs family and play important roles in plant growth, development, and the stress response (Afzal et al., 2008; Wrzaczek et al., 2010; Tanaka et al., 2012). Thus, negative ABA-signaling mediated by *CRK45* may play a specific and important role in the drought response of the tea plant.

Increased *PME41* expression was observed in the tea plant in Drought and RecDrought but not in Cold. PME participates in pectin remodeling, which keeps cells from separating, maintains plasma membrane integrity, and prevents cellular leakage. However, distinct genotype-, species- or tissue-dependent mechanisms of temperature control of *PME* activity have been found (Le Gall et al., 2015). For example, the overexpression of *Arabidopsis PME5* and *PMEI3* resulted in softer and harder shoot apical meristem cell walls, respectively (Peaucelle et al., 2011). We suppose that the mechanism of demethylesterification of pectin may be more important for drought defense rather than for cold defense in the tea plant. Further studies with more cultivars are necessary to check the involvement of *PME41* in the cold response of the tea plant.

#### Genes Specifically Upregulated in Cold

The genes specifically upregulated to a higher level in Cold were *SnRK1*.*2, HXK1, HXK2, bHLH43, bHLH79, bHLH7*, and *bHLH93*, which suggests that these genes are conservative and may play vital specific roles in response to cold stress.

HXKs phosphorylate glucose and fructose and participate in sugar signal transduction by modulating the abundances of diverse gene transcripts and integrating stress response substrates, including ABA and ethylene (Yue et al., 2015). In cold stress, *HXKs* are more induced in tolerant tea cultivars than in susceptible ones (Yue et al., 2015; Li Y. et al., 2019), which is consistent with our results. Another signaling intermediate, *SnRK1*, is involved in Suc, G6P, and T6P sensing and plays an important role in the plant response to sugar starvation (Wang Y. et al., 2019). Yue et al. (2015) found that *CsSnRK1*.*2* was induced by cold in the tea plant, whereas *CsSnRK1*.*1* was not elevated, and *CsSnRK1*.*3* was sharply suppressed. In our study, these three genes were activated in Cold and RecCold, but none was induced in Drought. These results indicate that sugar signal transduction and phosphorylation are more important defense mechanisms for cold tolerance in tea plant than for drought tolerance. However, more genotypes must be examined to confirm this conclusion.

Among the nine studied *bHLH* genes, some were specifically upregulated in response to cold stress. Cui et al. (2018) studied the *bHLH* family and proposed the following stress-related members in tea plant: *CsbHLH007, CsbHLH012, CsbHLH021, CsbHLH043, CsbHLH045* (ortholog of *ICE2*), *CsbHLH079, CsbHLH093*, and *CsbHLH095*. In our study, some of the genes were specifically upregulated in Cold, namely, *bHLH93, bHLH79, bHLH43*, and *bHLH7*, and these may play an important specific role in cold defense in the tea plant. We also observed that *CsbHLH012, CsbHLH021, CsbHLH045*, and *CsbHLH095* were downregulated in tea in Cold and/or Drought or did not differ from the control (Figure 4). This contradiction with Cui et al. (2018) can be explained by the variance in stress conditions: we evaluated long-term stress responses, whereas they evaluated 24 h stress induction (Cui et al., 2018). It may be that the mentioned TFs are more strongly induced by short-term cold stress.

Other genes that were upregulated in both Cold and RecCold were *WRKY42, ZAT30, POD73, LEA2, LEA3, TLP1*, and *FLS2*. Among them, the LEA proteins protect plant metabolism against abiotic stresses, marshaling properties that include antioxidant activity, metal ion binding, membrane and protein stabilization, hydration buffering, and DNA and RNA interactions (Chen et al., 2019). They also play an important role in stress acclimation (Ling et al., 2016). Liu Y. et al. (2016) investigated a maize *LEA3* gene expressed in *E*. *coli* and reported enhanced tolerance to low temperature. In rice, the *LEA2, LEA3*, and *DHN* groups have been found to show strong responses to osmotic stress (Yu et al., 2016). Our results on the tea plant showed no enhanced expression of *LEA2* and *LEA3* in Drought or RecDrought; however, Cold and RecCold greatly induced expression of both genes, indicating that these two genes can have specific functions on regulating cold tolerance in the tea plant.

*WRKY42* and *ZAT30* (*CCCH*) are zinc-finger proteins involved in the ABA-mediated stress response. We observed specific upregulation of *WRKY42* and *ZAT30* during cold and recovery in the tea plant. The *WRKY* genes are involved in stress and hormone signaling (Phukan et al., 2016; Jiang et al., 2017) during the drought stress response (Wang et al., 2016) and cold response (Samarina et al., 2020) in the tea plant. *ZAT30* (*CCCH*) is a zinc finger protein that is involved in developmental processes, responses to cold and osmotic stress (Pi et al., 2018), and participates in signal transduction. Both of these TF families are of particular interest, as they are involved in various biotic/abiotic stress responses and in developmental/physiological processes (Jiang et al., 2015). Maybe further studies are needed to confirm the role of the both genes in tea plant.

*TLP* is another gene with no elevated expression in Drought but greatly induced in Cold and RecCold. It is a member of the *TLPs*, made up of five pathogenesis-related proteins that are responsive to biotic and abiotic stress. The previous results indicate potential applications of TLP for crop improvement through a genetic transformation with applications in both biotic and abiotic stress protection, with strong evidence for a role in the crosstalk between the stress types. Transgenic plants that overexpress the *TLP* gene in different plant crops showed resistance to pathogens and tolerance to salinity and drought (Jesus-Pires et al., 2019). Our data confirm the possibly important role of *TLP1* in the cold stress response and in recovery in tea plant.

*FLS2*, which encodes receptor-like protein kinase, was also highly upregulated during Cold and RecCold in our study. *FLS2* is representative of the *RLK* family, playing an important role in mediating early flagellin signaling (Lu et al., 2010) upregulated in the cold response and recovery to stress. Our results are consistent with those of Li Y. et al. (2019), who found that *FLS2* exhibited a higher level of expression in tolerant tea cultivars with many-fold change under cold. We also speculate that plant-pathogen-related immunity mediated by *FLS2* may be important specifically for cold tolerance rather than for drought tolerance.

*PRP* and *GRP*, are covalently linked with pectin or hemicellulose and thus contribute to the strengthening of the cell wall in response to abiotic stress (Hijazi et al., 2014). In our study, significantly elevated expression of both *GRP* and *PRP* was observed in response to cold stress, which could indicate that the cell wall strengthening through pectin remodeling may be an important mechanism of tea plant cold tolerance. Earlier investigations also showed that one of the specific mechanisms of cold response in plants is the strengthening the cell wall, in contrast to the drought response (Beck et al., 2007).

#### Genes Upregulated at Recovery Treatments

Interestingly, some stress-inducible genes were seen to have higher transcript abundance during the recovery stages than had been seen in previous stress treatments. In RecCold, these genes were *AP-ERF-AP, LEA3, GST, SnRK1*.*3, SUS1, CIP, EGASE11*, and *TLP1*. In RecDrought, they were *WRKY42, CBF1, DREB26, ZAT, PEI54, CIP, CRK45*, and *PME41*. This indicates that the recovery of the tea plant after stress is a complex process and is important for defensive responses, and its regulation pathways differ from those for Cold and Drought. Moreover, we observed that RecCold and RecDrought produce very different responses, and *CIP* was the only gene upregulated in both recovery treatments in the tea plant. *CIP* belongs to the dehydrin family, and functional predictions suggest that this protein protects the membranes and prevents macromolecular coagulation or sequestration of calcium ions by association or disassociation with membrane under low-temperature conditions (Liu et al., 2006). We conclude that this gene may have a specific important function in recovery in tea plant.

Among the upregulated transcription factors, *AP-ERF-AP, DREB26*, and *CBF1* are representatives of the *AP2/ERF* family and mediate the transcriptional regulation of osmotic stress-responsive genes (Licausi et al., 2013; Parmar et al., 2019). Our results demonstrate that these genes are not only involved in the stress response but also in the recovery of the tea plant after cold and drought stresses. Previous gene expression studies have reported that most *AP2/ERFs* are expressed at low levels under normal conditions, but their expression can be induced or repressed at certain growth stages by hormones and stress stimuli (Xie et al., 2019). The *DREB* subfamily may be a key candidate for future exploration of a means to enhance drought and cold tolerance in tea (Ban et al., 2017; Parmar et al., 2019; Wang et al., 2019; Hu et al., 2020). It has been classified into six subgroups (A1–A6) (Sakuma et al., 2002). Among these, *DREB08* and *DREB26*, the A5 subgroup, encode repressor proteins inhibiting the expression of other *DREB* TFs (Dong and Liu, 2010). This means that they can suppress defense and stress-inducible genes in the absence of stress. In our study, increased expression of *DREB26* was observed during drought stress and also during recovery. This partly contradicts previous results that indicated that transcription levels of *DREB26* were hardly changed under drought and cold in *Vitis vinifera* (Zhao et al., 2014). Further studies of this gene in the tea plant are necessary for a comprehensive understanding of its role in stress responses.

Out of the other studied genes, with the pronounced expression profile during Recovery two genes are related to the cell wall remodeling, these are *EGases* and *PEI*. EGases are important cell wall-related proteins that modulate cell wall extensibility, which mediates cell enlargement and expansion. The *EGase11* gene in the tea plant was significantly upregulated in RecCold, Drought, and RecDrought, indicating cellulase growing activity. This result is not easy to explain, and further investigation is necessary. Earlier studies of these genes reported that increased hydrolases activity is evidence of cell wall degradation (Le Gall et al., 2015). PEIs are invertase inhibitor-related proteins and play an important role in the regulation of metabolic enzymes and viscoelastic properties of the cell wall (Wu et al., 2010). In our study, the elevated expression of PEI54 observed in Cold, RecCold, and Rec Drought indicates that pectin methylesterification in cell walls is activated in these stress treatments. These results showed that the cell wall remodeling activity is enhanced not only during the stress response but also during the recovery in tea plant. The genes encoding the cell wall remodeling enzymes can be further studied more comprehensively in tea plant as they might play a very important role in the responses to abiotic stresses and recovery after stress.

### Correlations in Different Responses

Based on expression profiles we tried to find correlations between responses to drought and cold. Highly correlated gene modules with specific expression patterns can help illustrating the framework of stress transcriptome. This analysis provides evidences about common and unique stress mechanism components under cold and drought stress in *C. sinensis*. In *A. thaliana* gene co-expression network analysis revealed 21 and 16 highly inter-correlated gene modules with specific expression profiles under drought and cold stress respectively (Sharma et al., 2018). In oil palm the significant correlations were found between cold-responsive genes and physiological parameters that helped to better understand the regulation networks (Li J. et al., 2019).

In our study, six genes (*DHN3, bHLH79, PEI54, SnRK1*.*2, SnRK1*.*3*, and *Hydrolase22*) were correlated positively and four genes (*CBF1, GOLS1, HXK2*, and *HXK3)* were correlated negatively in response to Cold and Drought. This indicated that the mentioned genes have the similar expression character during cold and drought. Under drought induction in tea plant we found twelve genes (*POD73, bHLH79, AP-ERF-ERF, LEA3, Hydrolase 22, SnRK1*.*2, CRK45, BMY5, bHLH93, bHLH95, DREB26*, and *HXK2)* that were positively correlated and three genes (*DHN1, CBF1*, and *GOLS1)* that were negatively correlated between Drought and RecDrought. On the other hand, under cold induction in tea plant we found six genes (*bHLH79, AP-ERF-ERF, LEA3, PRP, HXK1*, and *TLP1)* that were positively correlated and three genes (*SnRK1*.*1, SUS1*, and *DHN1*) that were negatively correlated between Cold and RecCold. Based on these results it can be speculated that recovery stage after drought is more similar to Drought response than RecCold–to Cold response.

In general, our results showed that more genes were activated in response to cold rather than drought in tea plant. These results corresponds with the transcriptomic studies reported that much more DEGs were upregulated under cold rather than drought in tea plant (Zheng et al., 2016), apple (Li X. et al., 2019) and in maize (Lu et al., 2017). Cold induces an extensive activation of transcription, drought stress, however, induced fewer transcriptional changes (only 15% as many), than cold in maize (Lu et al., 2017) suggesting that the more sensitive response to cold rather than drought would be a conserved mechanism in many plant species.

In other studies, an overlap between the expression patterns of stress-responsive genes in several plant species was observed after drought and cold stress induction (Li X. et al., 2019). In apple they found evidence of crosstalk between drought and cold stress signaling, with 377 commonly upregulated and 211 commonly downregulated genes (Li X. et al., 2019). In tomato, only about 10% of the drought-inducible genes were also induced by cold indicating different molecular strategies in their reaction to the two stresses (Zhou et al., 2019). In maize, only 194 DEGs were shared in cold and drought and, nearly 90% among them are regulated in a similar manner by both stresses, indicating that there is a shared network to regulate the cold and drought induced responses (Lu et al., 2017). On the other hand, specific regulations in response to cold or drought were also clearly visible in these crops. Nevertheless, in some plant species, the induction of cold resistance also promotes drought resistance and high-salinity tolerance, which is consistent with an increase in the levels of osmo-regulatory compounds and antioxidant enzyme activities (Hossain et al., 2013).

The effects of drought and cold reported here have arisen from a limited range of potential types and severities of stress. A greater range of treatments for (e.g., timing, severity, frequency) need to be examined in future studies to provide more clues for understanding the adaptation and tolerance mechanisms in tea plant.

## Conclusion

Using an *in silico* approach combined with an experimental approach, we confirmed the involvement of the nine new genes in the cold and/or drought response of tea plant: *GOLS1, GOLS3, GR-RBP3, HYDROLASE22, RHL41, CAU1, PME41, DREB26*, and *CRK45*. We hypothesized that many genes have similar expression profiles between the cold and drought responses of the tea plant. However, of 45 genes studied, only ten were significantly upregulated in response to both cold and drought: *HSP70, GST, SUS1, DHN1, BMY, bHLH102, GR-RBP3, ICE1, GOLS1*, and *GOLS3*. These genes can be considered as genes of non-specific stress response. *SnRK1*.*2, HXK1*/*2*, and *bHLH7*/*43*/*79*/*93* were upregulated in response to cold only, and the expression levels of *RHL41, CAU1*, and *Hydrolase22* were increased in the drought response. Interestingly, we found that the expression of *CIP* was higher in the recovery stage of both stresses, indicating its potentially important role in plant recovery after stress. In addition, some genes, such as *DHN3, bHLH79, PEI54, SnRK1*.*2, SnRK1*.*3*, and *Hydrolase22*, were significantly positively correlated between the cold and drought responses. *CBF1, GOLS1, HXK2*, and *HXK3*, by contrast, showed significantly negative correlations between the cold and drought responses. Because overexpression of many new candidate genes can confer stress tolerance, these proteins may play a promising role in agriculture in the context of plant genetic engineering. The isolation, cloning, characterization, and functional validation of novel candidate genes in response to diverse stress conditions are expected to be growth areas of research in coming years. In addition, the identification of the interaction partners of these proteins and the factors affecting these interactions is necessary to understand their role in conferring protection against different stress conditions in tea plants. These results provide valuable information and robust candidate genes for future functional analyses to improve the stress tolerance of the tea plant.

## Data Availability Statement

The datasets presented in this study can be found in online repositories. The names of the repository/repositories and accession number(s) can be found in the article/Supplementary Material.

## Author Contributions

LS planned and conducted experimental part of the manuscript, analyzed data, and wrote the manuscript. AB and AD performed bioinformatics part of the manuscript. LM participated in statistical analyses and critically reviewed the manuscript. AM, RR, and NK performed phenotyping and gene expression analyses. KM helped in gene-expression protocols improvement. VM, AR, WT, EX, and YO are scientific consultants and critically revised the manuscript. All authors contributed to the article and approved the submitted version.

## Conflict of Interest

The authors declare that the research was conducted in the absence of any commercial or financial relationships that could be construed as a potential conflict of interest.
